# Reversion to ancestral Zika virus NS1 residues increases competence of *Aedes albopictus*

**DOI:** 10.1371/journal.ppat.1008951

**Published:** 2020-10-14

**Authors:** Lili Kuo, Anna S. Jaeger, Elyse M. Banker, Sean M. Bialosuknia, Nicholas Mathias, Anne F. Payne, Laura D. Kramer, Matthew T. Aliota, Alexander T. Ciota

**Affiliations:** 1 The Arbovirus Laboratory, Wadsworth Center, New York State Department of Health, Slingerlands, NY, United States of America; 2 Department of Veterinary and Biomedical Sciences, University of Minnesota, Twin Cities, St. Paul, MN, United States of America; 3 Department of Biomedical Sciences, State University of New York at Albany School of Public Health, Albany, NY, United States of America; The University of Chicago, UNITED STATES

## Abstract

Both mosquito species-specific differences and virus strain -specific differences impact vector competence. Previous results in our laboratory with individual populations of N. American mosquitoes support studies suggesting *Aedes aegypti* are more competent than *Ae*. *albopictus* for American Zika virus (ZIKV) strains and demonstrate that U.S. *Ae*. *albopictus* have higher competence for an ancestral Asian ZIKV strain. A982V, an amino acid substitution in the NS1 gene acquired prior to the American outbreak, has been shown to increase competence in *Ae*. *aegypti*. We hypothesized that variability in the NS1 could therefore contribute to species-specific differences and developed a reverse genetics system based on a 2016 ZIKV isolate from Honduras (ZIKV-WTic) to evaluate the phenotypic correlates of individual amino acid substitutions. In addition to A982V, we evaluated G894A, which was acquired during circulation in the Americas. Reversion of 982 and 894 to ancestral residues increased infectivity, transmissibility and viral loads in *Ae*. *albopictus* but had no effect on competence or replication in *Ae*. *aegypti*. In addition, while host cell-specific differences in NS1 secretion were measured, with significantly higher secretion in mammalian cells relative to mosquito cells, strain-specific differences in secretion were not detected, despite previous reports. These results demonstrate that individual mutations in NS1 can influence competence in a species-specific manner independent of differences in NS1 secretion and further indicate that ancestral NS1 residues confer increased competence in *Ae*. *albopictus*. Lastly, experimental infections of *Ifnar1*^*-/-*^ mice demonstrated that these NS1 substitutions can influence viral replication in the host and, specifically, that G894A could represent a compensatory change following a fitness loss from A982V with some viral genetic backgrounds. Together these data suggest a possible role for epistatic interactions in ZIKV fitness in invertebrate and vertebrate hosts and demonstrate that strains with increased transmission potential in U.S. *Ae*. *albopictus* could emerge.

## Author summary

The factors that facilitated the emergence and rapid dissemination of ZIKV in the Americas, and the specific role of strain variation and adaptive evolution in ZIKV transmission, are not fully understood. It has been shown that an amino acid substitution in the NS1 gene acquired prior to the American outbreak confers increased infectivity in *Ae*. *aegypti*. Therefore, we hypothesized that variability in the NS1 could contribute to species-specific differences. To investigate the role of the ZIKV NS1 in competence of both *Ae*. *aegypti* and *Ae*. *albopictus* we utilized reverse genetics to revert to the ancestral NS1 residues and characterized the resultant mutant strains. Reversions increased infectivity, transmissibility and viral loads in *Ae*. *albopictus* but had no effect on competence or replication in *Ae*. *aegypti*. Additionally, we assessed the effect of these mutations on NS1 secretion and both virulence and viremia kinetics using a ZIKV mouse model. NS1 secretion, as well as vertebrate morbidity or mortality, were similar among strains, yet strain-specific differences in viremia levels were identified. These results demonstrate that individual mutations in NS1 can influence competence in a species-specific manner independent of differences in NS1 secretion and further indicate that ancestral NS1 residues confer increased competence in *Ae*. *albopictus*. Moreover, contrasting these data with previous studies suggests a role for epistatic interactions in ZIKV fitness in invertebrate and vertebrate hosts.

## Introduction

Zika virus (ZIKV; *Flaviviridae*, *flavivirus*) was first isolated in Uganda in 1947 [1] and became a public health concern following an outbreak on the islands of Yap in 2007 [2]. Rapid expansion across the globe occurred thereafter with ZIKV outbreaks in French Polynesia (2013) and the surrounding islands of the South Pacific (2014) [3]. Autochthonous transmission of ZIKV was first identified in the Western Hemisphere in Brazil in May 2015 [4] but isolates from 2014 from Haiti have since been identified [5]. ZIKV subsequently expanded throughout Latin America and into the U.S. and, although infections have decreased dramatically since 2017, there were over 800,000 suspected or confirmed cases occurring in at least 48 Western countries from 2015 to 2018 [6]. Sequence analyses indicate that there are two distinct phylogenetic lineages of ZIKV; African and Asian [7,8]. It has been shown that a virus belonging to the Asian lineage was the source of the outbreak in the Americas [9].

Globally, *Ae*. *aegypti* is the primary vector for ZIKV, while *Ae*. *albopictus* is considered to be a secondary vector and other species have been implicated [10]. Both species have been shown to be competent vectors of ZIKV in experimental settings [11–14], although transmission rates are generally higher for *Ae*. *aegypti* and can vary with both population and virus strain [15]. Previous studies support the idea that *Ae*. *aegypti* have higher transmission rates for American ZIKV [16] but also suggest that ancestral strains may have increased competence in *Ae*. *albopictus* [15]. As documented with West Nile virus (WNV) [17] and chikungunya virus (CHIKV) [18–21], viral adaptation to mosquito vectors can drive transmission and facilitate rapid geographic expansion [22]. Liu et al. investigated the phenotypic effects of many new world ZIKV mutations and found that a single mutation in the ZIKV NS1 gene, resulting in an alanine to valine amino acid change at position 982 in the polyprotein (position 182 in the NS1), increases competence in *Ae*. *aegypti* and therefore may have contributed to the emergence and dispersal of ZIKV in the Americas [16].

We developed a ZIKV infectious clone based on a 2016 ZIKV isolate from Honduras (ZIKV HND; [15]). In addition to A982V, ZIKV HND has a glycine to alanine amino acid substitution at position 894 (position 94 in the NS1) acquired during circulation in the Americas and shared among recent Central American isolates. To investigate the role of the ZIKV NS1 in competence of both *Ae*. *aegypti* and *Ae*. *albopictus*, we utilized reverse genetics to revert to the ancestral NS1 residues and characterized the resultant mutant strains. Additionally, we assessed the effect of these mutations on virulence and viremia kinetics using an *Ifnar1*^*-/-*^ ZIKV mouse model. Our results highlight the species-specific nature of ZIKV interactions and the role of the ZIKV NS1 in viral fitness.

## Materials and methods

### Phylogenetic analysis

Seventy-seven representative sequences spanning

KY415986.1,KY325469.1, KX198135.2, KY415987.1, MF098771.1, MF574576.1, NC_035889.1, KY317936.1, KY693678.1, LC190723.1, KU922923.1, MF664436.1, MF574554.1, MF801381.1, KY558999.1, MH158237.1, KY785410.1, MF434517.1, MF801378.1, KY631494.1, KY075935.1, KX922705.1, MF593625.1, KX811222.1, KY693679.1, KY325464.1, MF801384.1, KY014296.2, MF438286.1, MG494697.1, KY765325.1, KU647676.1, MF098768.1, KY075934.1, KX702400.1, KJ776791.2, KY325467.1, KY559005.1, KY693676.1, KX056898.1, KY325465.1, KY075939.2, KY648934.1, MF384325.1, KX879603.1, KX694534.2, KX601168.1, KX269878.1, MF574583.1, MG595216.1, MF801396.1, MH157202.1, MH157213.1, MF159531.1

KY785484.1, KY785423.1, KY785445.1, KY785419.1, KY785426.1, MH063265.1, KY559021.1, KX694532.2, MH158236.1, KY785414.1, KY785462.1, MF434518.1, KY785424.1, KY785413.1, KY559001.1, KY785451.1, MH063259.1, KY325471.1, KY785457.1, KY325466.1, KY348860.1, KX601166.2, MH130094.1

Alignment, model testing, and phylogenetic analysis of consensus sequences were completed with the CLC Genomics Workbench. A general reversible substitution model was used [9] and phylogeny was inferred using Maximum Likelihood methods with 500 replicates. The tree was rooted to MR766 (GenBank accession no. KX421193.1) and edited in FigTree v1.4.2 (http://tree.bio.ed.ac.uk/software/figtree).

### Infectious clone development

A ZIKV infectious clone (ZIKV WTic) was developed from ZIKV HND 2016 (GenBank Accession no. KX906952). The strain was isolated by the New York State Department of Health (NYSDOH) Arbovirus Laboratory from patient serum following travel to Honduras in early 2016. Stock virus was created with one additional passage on C6/36 mosquito cell culture (CRL-1660; ATCC, Manassas, VA, USA).

Viral RNA was extracted (RNeasy Mini Kit, QIAGEN) from virus stock harvested from the first passage on C6/36 cells. For construction of the full-length cDNA clone, four overlapping cDNA fragments spanning the entire viral genome were synthesized from purified viral RNA (SuperScript III Reverse Transcriptase Kit, Invitrogen) and ZIKV-specific primers. For the generation of a reverse genetic system, low-copy number plasmid pACYC177 (ATCC, Manassas, VA, USA) was chosen as the backbone. The vector was modified between the XhoI and ClaI sites with a synthesized 490-bp DNA fragment (Integrated DNA technologies, Coralville, IA, USA) containing a T7 promotor sequence, unique restriction sites for sequential insertion of viral cDNA fragments, and the canonical Hepatitis D virus (HDV) ribozyme sequence [23]. The cDNA fragments from reverse transcription (RT)-PCR were cloned into the modified plasmid using NEBuilder HiFi DNA assembly system (New England Biolabs, Ipswich, MA, USA). As a measure to manage the toxicity of the NS1 gene [24], the reverse genetics system was designed to contain two separate plasmids (p): pZika(Hond)-ABD and pZika(Hond)-NS1. Plasmid pZika(Hond)-ABD was propagated in E. coli 10-beta cells (New England Biolabs, Ipswich, MA, USA) and it encompasses the entire viral genome except the majority of the NS1 region which was replaced with a NotI restriction site. A 965-bp fragment of the NS1 region flanked with EcoRI sites was cloned separately as pZika(Hond)-NS1, and the plasmid was transformed and maintained in E. coli Stable cells (New England Biolabs, Ipswich, MA, USA). Both plasmids were verified by restriction analysis and DNA sequencing in both orientations.

**Fig 1:**
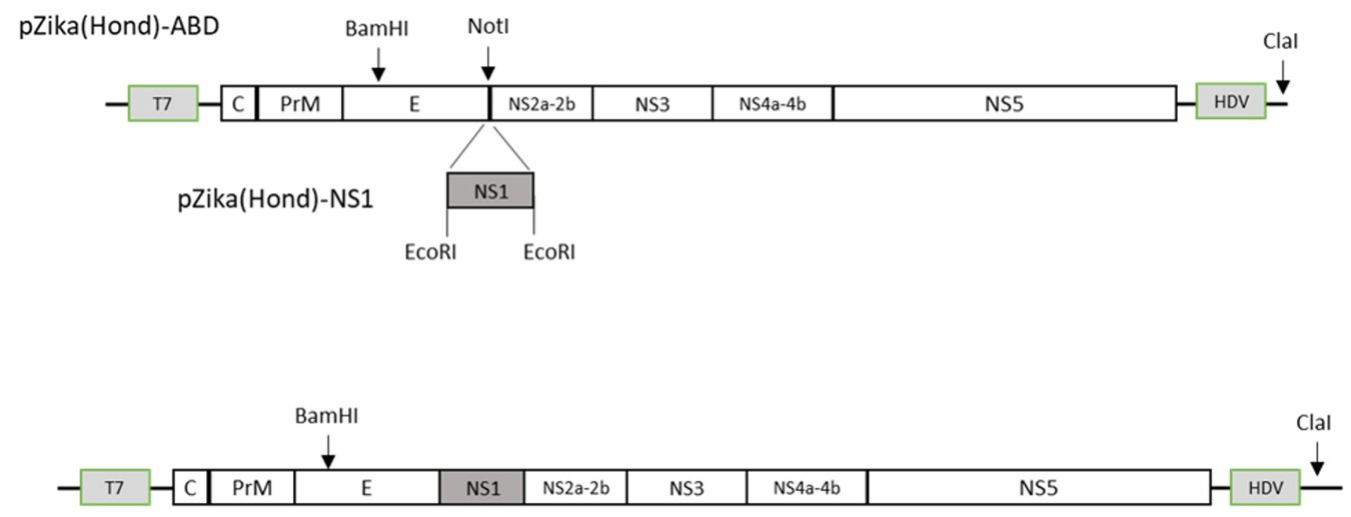
Generation of a ZIKV full-length reverse genetics system. A low-copy number plasmid (pACYC177) was modified between the XhoI and ClaI restriction sites to include a 490-bp fragment containing the T7 promoter sequence, unique restriction sites and the HDV ribozyme sequence. cDNA fragments spanning the entire ZIKV genome were synthesized from stock viral RNA and cloned into the modified plasmid. Two separate constructs were utilized to manage the toxicity of the NS1 region: pZika(Hond)-ABD and pZika(Hond)-NS1. Plasmid pZika(Hond)-ABD encompasses the entire ZIKV genome, but lacks the majority of the NS1 region, which is replaced with a NotI restriction site. Plasmid pZika(Hond)-NS1 contains a 965-bp fragment of the NS1 region flanked with EcoRI restriction sites. To distinguish between rescued and parental virus, genetic markers were placed into the cDNA clone within the E gene to eliminate the AvrII restriction site while simultaneously generating a BamHI restriction site.

A genetic marker was engineered into the cDNA clone to distinguish between rescued recombinant virus and the parental virus (Fig 1). Three silent nucleotide changes were placed at positions 1527–1535 in the E gene to abolish the AvrII site while simultaneously creating a BamHI site.

### *In vitro* transcription and transfection

To generate the full-length template for *in vitro* transcription, pZika(Hond)-ABD was linearized with NotI and the EcoRI-digested NS1 region was assembled into the larger plasmid using NEBuilder DNA assembly master mix (New England Biolabs, Ipswich, MA, USA). The assembled, full-length cDNA was purified using DNA Clean & Concentrator-5 columns (Zymo Research, Irvine, CA, USA), digested with ClaI restriction enzyme (New England Biolabs, Ipswich, MA, USA) and subsequently purified again to generate a linearized DNA template for in vitro transcription. Capped, runoff RNA transcripts were synthesized using Megascript T7 transcription kit (Thermo Fisher, Waltham, MA, USA), supplemented with 2mM Anti-Reverse Cap Analog (ARCA; New England Biolabs, Ipswich, MA, USA), as specified in manufacturer’s protocol. Recombinant viruses were generated by RNA transfection as described previously [25]. Briefly, in vitro synthesized genomic viral RNA, without further purification, was transfected into 10^7^ Vero cells in 0.8 mL of cold phosphate-buffered saline (PBS), pH 7.4, via electroporation by three pulses at 0.85 kV and 25 μF using a GenePulser apparatus (Bio-Rad Laboratories, Hercules, CA, USA). Transfected cells were plated in a T-75 flask in media containing 10% fetal bovine serum (FBS) and incubated at 37°C and 5% CO2 until cytopathic effect (CPE) was observed.

Rescued recombinant viruses were harvested 5–7 days post-transfection depending on the extent of CPE, and then passed once (P1) on C6/36 cells. To ensure no contaminate input RNA was present in rescued viruses the engineered genetic markers of AvrII and BamHI were analyzed in P1 virus stock by extracting viral RNA (RNeasy Mini Kit, QIAGEN) and subsequently subjecting it to RT- PCR followed by restriction digest analysis. A DNA fragment of 462-bp encompassing the genetic marker was amplified with primers Hond-F12 (5’-GAAATGGATGTGGACTTTTTGGCAAAGGG-3’) and Hond-R8 (5’-GACAGTTTGCCTTTTGGCATGTGCG-3’). The DNA fragment was subsequently purified using DNA Clean and Concentrator system (Zymo Research, Irvine, CA, USA), digested with either BamHI or AvrII enzymes and analyzed on a 1.5% agarose gel.

### Site-directed mutagenesis

ZIKV-V982A, ZIKV-A894G and a double-mutant containing both mutations (ZIKV -NS1dm), were developed using pZIKA-NS1 as the scaffold for construction. The mutations were generated via splicing by overlap extension (SOE) PCR [26]. For mutation A894G, the codon GCT for Ala was changed to GGG for Gly, and for mutation V982A the codon GTT for Val was changed to GCC for Ala.

After sequence confirmation, the full-length cDNA template was assembled using NEBuilder HiFi DNA assembly system (New England Biolabs, Ipswich, MA, USA) and prepared for *in vitro* RNA transcription and transfection in Vero cells as previously described. Cytopathic effect (CPE) was monitored daily. The virus was harvested between day 5 and day 7 post infection (pi), depending of the extent of CPE, and was stored in a final concentration of 20% FBS at -80°C (designated as P0). Viral RNA was extracted (RNeasy Mini Kit, QIAGEN), amplified by RT-PCR (SuperScript III Reverse Transcriptase Kit, Invitrogen) and sent for Sanger sequencing at the WCAGTC (Albany, NY, USA).

Virus with confirmed sequence was amplified in C6/36 cells seeded in T-125 flasks. After a one-hour absorption period at 28°C C6/36 the appropriate maintenance media was added to each flask. Virus was then held at 28°C for 7 days, while CPE was monitored daily. Virus was harvested in a final concentration of 20% FBS and was stored at -80°C. Viruses were designated according to the mutation which they contain (ZIKV-V982A, ZIKV-A894G; ZIKV-NS1dm [V982A/A894G]). Sequences of all viruses were confirmed following cell culture amplification.

### Virus titration

Stock virus was titrated by plaque assay on Vero cells according to [27]. Virus was serially diluted in bovine albumin (BA)-1 cell culture media. Vero cells were inoculated with 100 μL of each dilution in duplicate and given a one-hour adsorption period at 37°C. A 1:1 solution of 2% oxoid agar and EMEM 10% FBS was used for the first overlay. The plates were held at 37°C for 5 days, at which time the second overlay was applied (1:1 solution 2% oxoid agar and EMEM 2% FBS plus 2 mL neutral red/100 mL agar/EMEM solution). Plaques were counted on day 6, and mean titers were calculated and compared to ZIKV-WTic by t-test.

### *In vitro* growth kinetics

Confluent monolayers of human alveolar epithelial (A549, ATCC, Manassas, VA, USA) cells, Vero cells and C6/36 cells were inoculated with ZIKV strains in duplicate at a multiplicity of infection (MOI) of 0.01 plaque forming units (PFU) per cell. After a one-hour absorption period at 37°C (Vero and A549) or 28°C (C6/36), the inoculum was removed, and cells were washed twice with appropriate maintenance media. Cultures were maintained in 6-well plates with 3 mL of maintenance media and incubated at 37°C (Vero and A549) or 28°C (C6/36). Samples of 100 μl supernatant were harvested at days 1–4 (Vero and A549) or 1–7 (C6/36) pi, diluted 1:10 in media containing 20% FBS, and stored at -80°C. Titrations were performed in duplicate by plaque assay on Vero cells [27] and mean titers for each time point were calculated. Growth kinetics were compared using repeated measured ANOVA and Tukey’s post hoc tests (GraphPad Prism, Version 5.0).

### Mouse infection experiments

This study was approved by the University of Minnesota, Twin Cities Institutional Animal Care and Use Committee (Protocol Number 1804–35828). *Ifnar1*^*-/-*^ mice on the C57BL/6 background were bred in the specific pathogen-free animal facilities of the University of Minnesota, Twin Cities. Randomized, mixed-sex groups of three- to seven-week-old mice were subcutaneously inoculated in a hind footpad with 10^3^ PFU of ZIKV-WTic, ZIKV-NS1dm, ZIKV-A894G, and ZIKV-V982A in a volume of 20 μL. Mice were weighed and monitored daily for clinical signs of disease for 21 days. Sub-mandibular blood draws were performed at 2- and 4-days-post-inoculation (dpi) and serum samples were collected to compare viremia, and to measure NS1 concentrations. Mice were humanely euthanized at the end of experimentation at 21 dpi, or when mice met euthanasia criteria prior to experimental endpoint—greater than 20% starting weight lost or a clinical score greater than 3. Mouse infections were conducted in two independent experimental replicates for a total of n = 8–11 mice per infection group.

### Vector competence

*Ae*. *aegypti* were collected in Poza Rica, Mexico in 2016 and *Ae*. *albopictus* were collected in Suffolk County, NY in 2014. Each population was subsequently colonized at the Arbovirus Laboratory, Wadsworth Center, New York State Department of Health (NYSDOH) according to standard rearing protocol [28].

Four to 7-day old female mosquitoes were deprived of sucrose for 18–24 hours and offered blood meal mixtures using a Hemotek membrane feeding system (Discovery Workshops, Acrington, UK) with a porcine sausage casing membrane. For all preliminary and experimental feedings, the blood meals consisted of defibrinated sheep blood (Colorado Serum Co., Denver, CO, USA) with 2.5% sucrose, frozen stock virus and a final concentration of 10% sodium bicarbonate. Zika virus strains (ZIKV HND 2016, ZIKV WTic, ZIKV-V982A, ZIKV-A894G and ZIKV-dm) were diluted in C6/36 maintenance media to achieve a titer of 7.0 log_10_ PFU/mL. For all blood feeding experiments, mosquitoes were sedated with CO_2_ following one hour of feeding and fully engorged mosquitoes were transferred to 0.6L cartons and maintained at 27°C for experimental testing. Infection, dissemination, and transmission rates were determined as previously described [29] on day seven or 14 post-feeding (pf). Twenty-four to 60 (*Ae*. *aegypti*) and 28–87 (*Ae*. *albopictus*) mosquitoes were sedated with trimethylamine [TEA, (Sigma-Aldrich, St. Louis, MO, USA)] and legs were removed and placed in 1 mL mosquito diluent [MD; 20% heat-inactivated fetal bovine serum (FBS) in Dulbecco’s phosphate-buffered saline (PBS) plus 50 μg/mL penicillin/streptomycin, 50 μg/mL gentamicin, and 2.5 μg/mL fungizone]. Mosquitoes were allowed to expectorate for 30 minutes into capillary tubes charged with ~20 μL FBS plus 50% sucrose (1:1), at which time the mixture was ejected into 250 μL MD. Mosquito bodies were then placed into individual tubes with 1 mL MD. All samples were held at -80°C until tested. Bodies, legs and salivary secretions were processed and screened by ZIKV-specific qRT-PCR [30] to test for infection, dissemination and transmission, respectively. ZIKV body titers were calculated from standard curves based on infectious particle standards created from matched virus stocks. Data were analyzed using GraphPad Prism 4.0. Rates were compared with Fisher’s exact tests.

### NS1 detection by ELISA

Vertebrate (Vero) and mosquito (C6/36) cells were infected with ZIKV-WTic, ZIKV- V982A, ZIKV-A894G, and ZIKV-NS1dm at a multiplicity of infection (MOI) of 1.0 PFU/mL. Cell culture supernatant was removed at 72 (Vero) or 96 (C6/36) hours post-infection (hpi), representing peak titer in each cell line, and ZIKV NS1 Antigen ELISA Kit (ZIKV-NS1-EK, BioFront Technologies, Tallahassee, FL, USA) was used to determine the quantity of soluble NS1 (sNS1) protein secreted. Each sample was diluted according to the specifications using the sample dilution buffer provided. The standard curve was generated using the materials provided, and the quantities of ZIKV sNS1 were comparable to the linearity in the assay. The optical density (OD) was measured at 450 nm and extrapolated quantities were compared among strains by student’s t-tests.

## Results

### Phylogenetic analysis

**Fig 2:**
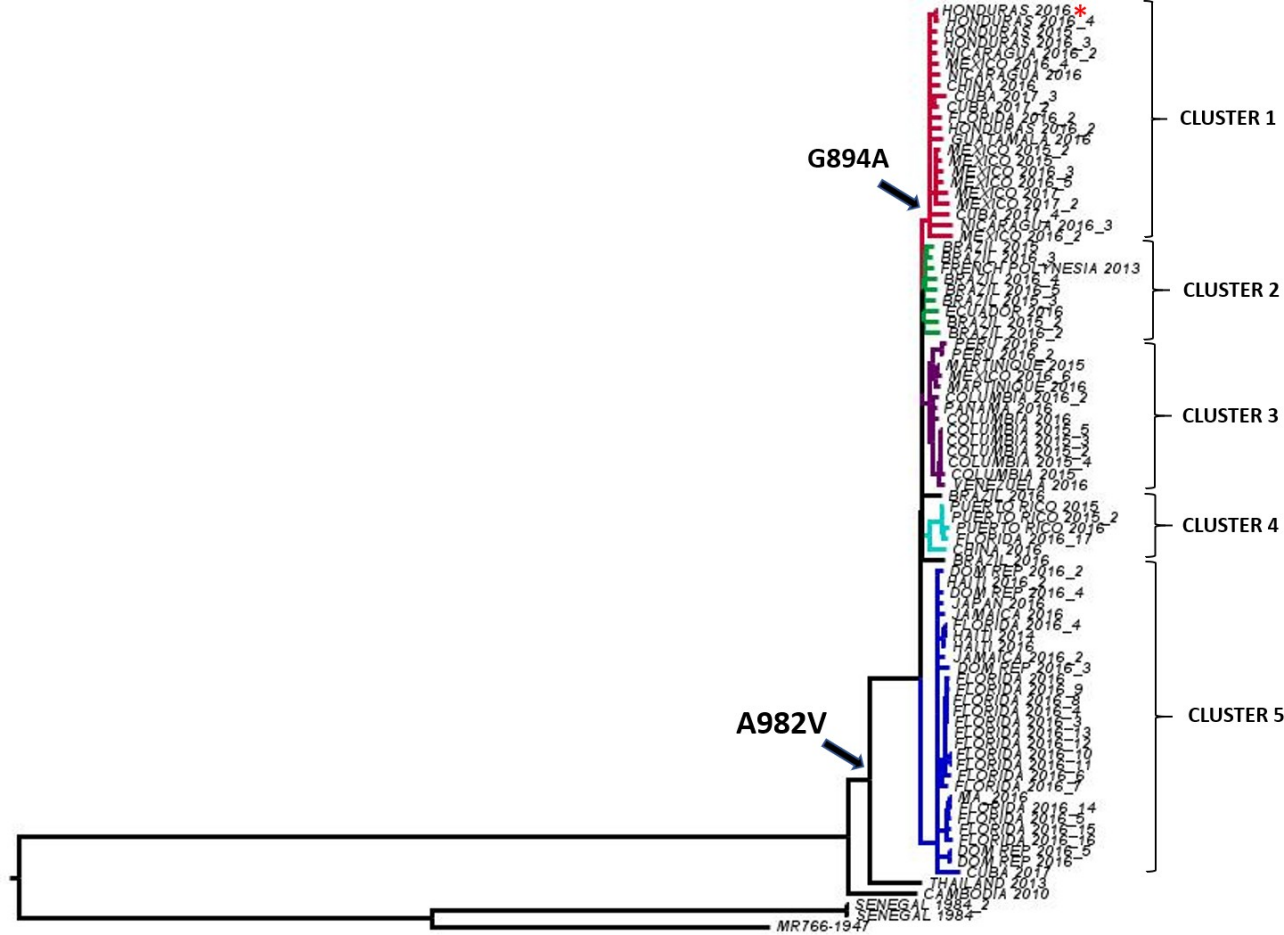
Phylogenetic analysis of Zika virus. A total of 77 ZIKV full-genome sequences were analyzed using a general reversible substitution model. Phylogeny was inferred using Maximum Likelihood methods with 500 replicates and the tree was rooted to ZIKV strain MR766 (GenBank accession no. KX421193.1). Arrows indicate the inferred occurrence of NS1 amino acid substitutions A982V and G894A. Well-supported clusters (bootstrap support >0.75) are indicated by unique colors. Cluster 1 contains the ZIKV HND 2016 strain (*) utilized in this study.

Phylogenetic analysis of 77 ZIKV sequences representing a sampling of geography and time was completed using a general reversible substitution model (Fig 2). Phylogeny was inferred using Maximum Likelihood methods with 500 replicates. The alanine to valine substitution at position 982 occurred prior to the introduction to the Americas and all strains from the Americas share it. The guanine to alanine substitution at position 894 occurred following the 982 substitution during ZIKV evolution in the Americas and is common to all strains in Cluster 1, including ZIKV HND 2016 (Fig 2). Strains most closely related to the initial introduction to the Americas, including the 2013 French Polynesian strain [31] form an independent cluster (Cluster 2, Fig 2). In all, there are 5 well-supported clusters, defined by bootstrap values > 0.75. While there is some temporal and geographic clustering, there is additionally evidence of strain dispersal. For instance, strains isolated in Florida, USA were identified in 3 of the 5 clusters, likely representing multiple introductions.

### *In vitro* growth kinetics

**Fig 3:**
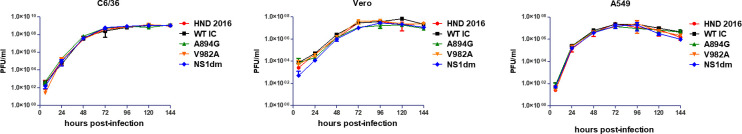
Equivalent growth kinetics of ZIKV strains in mosquito and mammalian cells. Mosquito (C6/36) and mammalian (Vero, A549) cells were infected with ZIKV strains at a multiplicity of infection of 0.01 PFU/cell in triplicate. Supernatant was harvested at each designated time point for titration by plaque assay. Overall kinetics and viral titers at individual timepoints were statistically equivalent (repeated measures ANOVA, p>0.05; Tukey’s multiple comparisons post-test, p>0.05).

In order to confirm that the replicative fitness of ZIKV-WTic was comparable to ZIKV HND 2016, and to assess replication of ZIKV-A894G, ZIKV-V982A, and ZIKV-NS1dm (A894G/V982A), multi-step growth kinetics experiments in mosquito (C6/36) and mammalian (Vero and A549) cells were completed. Peak viral titers were similar in mammalian cells (~7.5 log_10_ PFU/mL), with higher titers achieved in C6/36 cells (~9.0 log_10_ PFU/mL). There were no significant differences identified between ZIKV strains, both in overall kinetics (repeated measured ANOVA, F(4,30), p>0.05) or at individual time points (Tukey’s post-tests, p>0.05) in any cell line (Fig 3).

### Vector competence

In addition to *in vitro* experiments, a preliminary experimental feeding and competence study was completed to confirm that ZIKV-WTic was phenotypically equivalent to ZIKV HND 2016 in *Ae*. *aegypti*. Infection, dissemination and transmission rates were statistically equivalent on day 7 and 14 post-feeding (Fisher’s exact test, p>0.05; S1 Table).

Three independent ZIKV vector competence experiments were performed to compare ZIKV-WTic with ZIKV NS1 mutant strains in *Ae*. *aegypti* and *Ae*. *albopictus*. Although both species, all virus strains, and both time points were not tested in each independent experiment, those which were experimental replicates were statistically equivalent in terms of competence (Fisher’s exact test, p>0.05; S2 Table), and therefore combined for downstream comparative analyses. Replicate one included ZIKV-WTic and ZIKV-NS1dm in *Ae*. *aegypti* and *Ae*. *albopictus* assayed at both days, replicate two included ZIKV-NS1dm and ZIKV-V982A in *Ae*. *aegypti* assayed only at day 14, and replicate 3 included all strains in both species assayed at both time points (S2 Table). Sample sizes for individual strains and timepoints ranged from 24–60 for *Ae*. *albopictus* and 28–87 for *Ae*. *aegypti*. Stock viruses were diluted appropriately to achieve blood meal titers of 7.0 log_10_ PFU/mL and back titrations indicated a range from 6.6 to 7.2 log_10_ PFU/mL.

**Fig 4:**
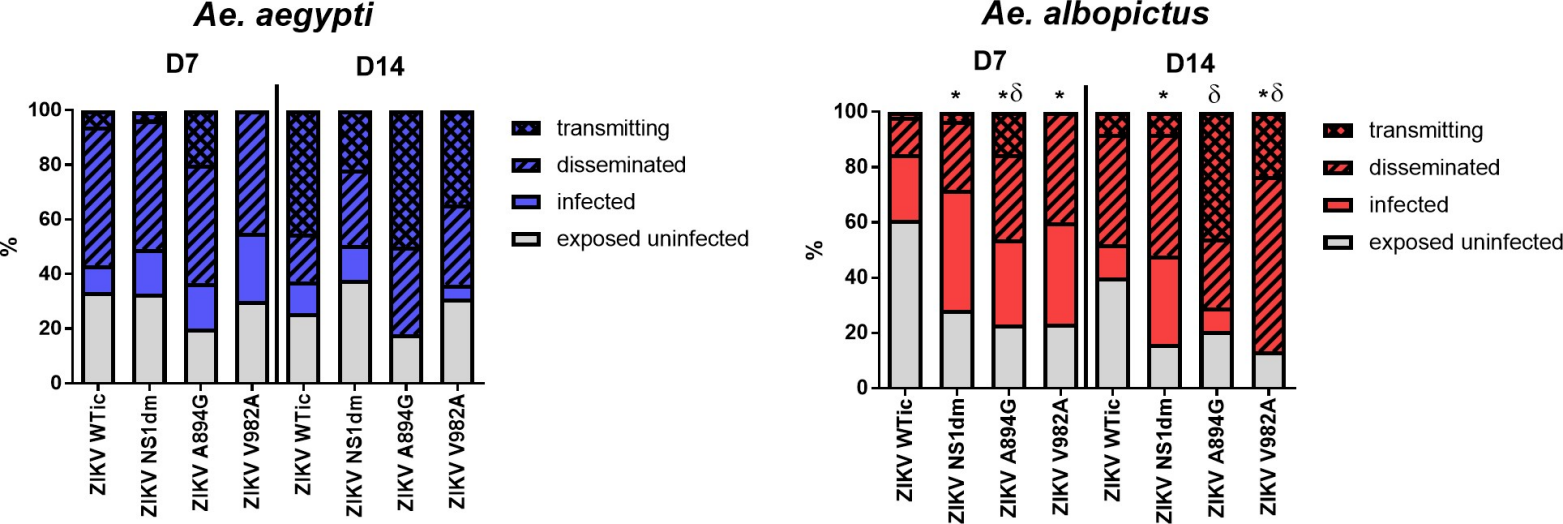
*Aedes albopictus* have increased vector competence for ZIKV strains with ancestral NS1. Mosquitoes were fed a blood meal with 6.6 to 7.2 log_10_ PFU/mL of various strains of ZIKV and were held for 7 or 14 days. Sample sizes for individual ZIKV strains and timepoints ranged from 24–60 for *Ae*. *albopictus* and 28–87 for *Ae*. *aegypti*. Infection, dissemination and transmission rates were determined by the number of qRT-PCR positive bodies, legs and salivary secretions, respectively. Statistically significant differences in infection rates (*) and transmission rates (δ) among exposed mosquitoes were identified for NS1 mutants relative to ZIKV WTic in *Ae*. *albopictus* (Fisher’s exact test, p<0.05).

**Fig 5:**
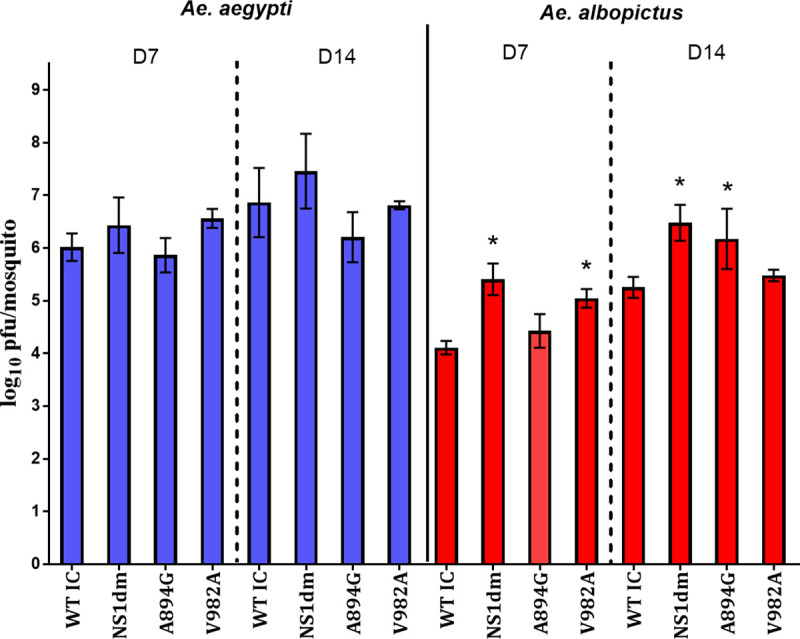
Higher viral loads of ZIKV NS1 mutants in *Ae*. *albopictus*. Zika virus quantities in mosquito bodies testing positive for virus were determined by qRT-PCR for *Ae*. *aegypti* and *Ae*. *albopictus* at 7 and 14 days post-feeding. A significant effect of mosquito species, ZIKV strain, and an interaction between the two was identified (two-way ANOVA, p<0.01). ZIKV-WTic body titers were significantly higher in *Ae*. *aegypti* relative to *Ae*. *albopictus* at both days andbody titers of NS1 mutants were significantly higher than ZIKV-WTic in *Ae*. *albopictus* (*two-way ANOVA, Tukey’s post-test, p<0.05).

There were no statistically significant differences measured between ZIKV-WTic and NS1 variants in *Ae*. *aegypti* when comparing the proportion of exposed infected, the proportion infected with disseminated infections, and the proportion infected transmitting virus, at day 7 or 14 post-feeding (Fig 4; Fisher’s exact test, p>0.05). In addition, no significant differences in viral load were measured in *Ae*. *aegypti* (Fig 5; two-way ANOVA, Tukey’s multiple comparison post-test, p>0.05). Conversely, differences in both vector competence and viral load were measured in *Ae*. *albopictus* (Figs 4 and 5). Specifically, infection rates were higher for ZIKV-NS1dm, A894G and V982A compared to ZIKV-WTic on both day 7 and 14 post-feeding, and these differences were statistically significant, with the exception of ZIKV-A894G on day 14 (Fig 4; Fisher’s exact test, p<0.05). Overall infection rates (day 7 and 14 combined) were 48.6% for ZIKV-WTic in *Ae*. *albopictus*, which is significantly lower than ZIKV-WTic in *Ae*. *aegypti* (70.6%; Fisher’s exact test, p<0.01). Overall infection rates for the 3 NS1 variants were statistically equivalent in *Ae*. *albopictus*, with a mean increase of 30% relative to ZIKV-WTic (i.e. mean infection rates of ZIKV-NS1dm, A894G and V982A were 78.6% in *Ae*. *albopictus*). The mean overall infection rate of NS1 variants in *Ae*. *aegypti* was similar to ZIKV-WTic (69.4%). Although proportions of infected mosquitoes with disseminated or transmitted virus were generally higher with NS1 variants in *Ae*. *albopictus*, the only statistically significant difference measured for transmission rates was with ZIKV-A894G, which was significantly higher than ZIKV-WTic on day 14 post-feeding (Fig 4; Fisher’s exact test, p<0.05). When considered as proportions of exposed, rather than infected, higher dissemination rates were measured on day 7 for ZIKV-A894G and V982A, and a higher transmission rate was measured on day 7 for ZIKV-A894G, relative to ZIKV-WTic (Fisher’s exact tests, p<0.05). On day 14, transmission rates were significantly higher for both ZIKV-A894G and V982A (Fisher’s exact test, p<0.05).

Results of virus quantification in mosquito bodies were similar to competence results. There was a significant effect of species (two-way ANOVA; DF = 1, MS = 8.32, F = 7.69, p = 0.0065) and ZIKV strain (DF = 3, MS = 10.52, F = 9.733, p<0.001), as well as an interaction between the two (DF = 3, MS = 6.87, F = 6.36, p = 0.005). ZIKV-WTic load in mosquitoes was significantly lower (~2 log_10_ PFU/ mosquito) in *Ae*. *albopictus* relative to *Ae*. *aegypti* at day 7 and 14 post-feeding (Fig 5; two-way ANOVA, p<0.001). Viral load was higher in *Ae*. *albopictus* infected with NS1 variants relative to ZIKV-WTic at both timepoints and these differences were significant for ZIKV- V982A and ZIKV-NS1dm at day 7 and ZIKV A894G and NS1dm at day 14 (Fig 5; two-way ANOVA, Tukey’s multiple comparison post-test, p<0.05).

### Secreted ZIKV NS1 quantification

**Fig 6:**
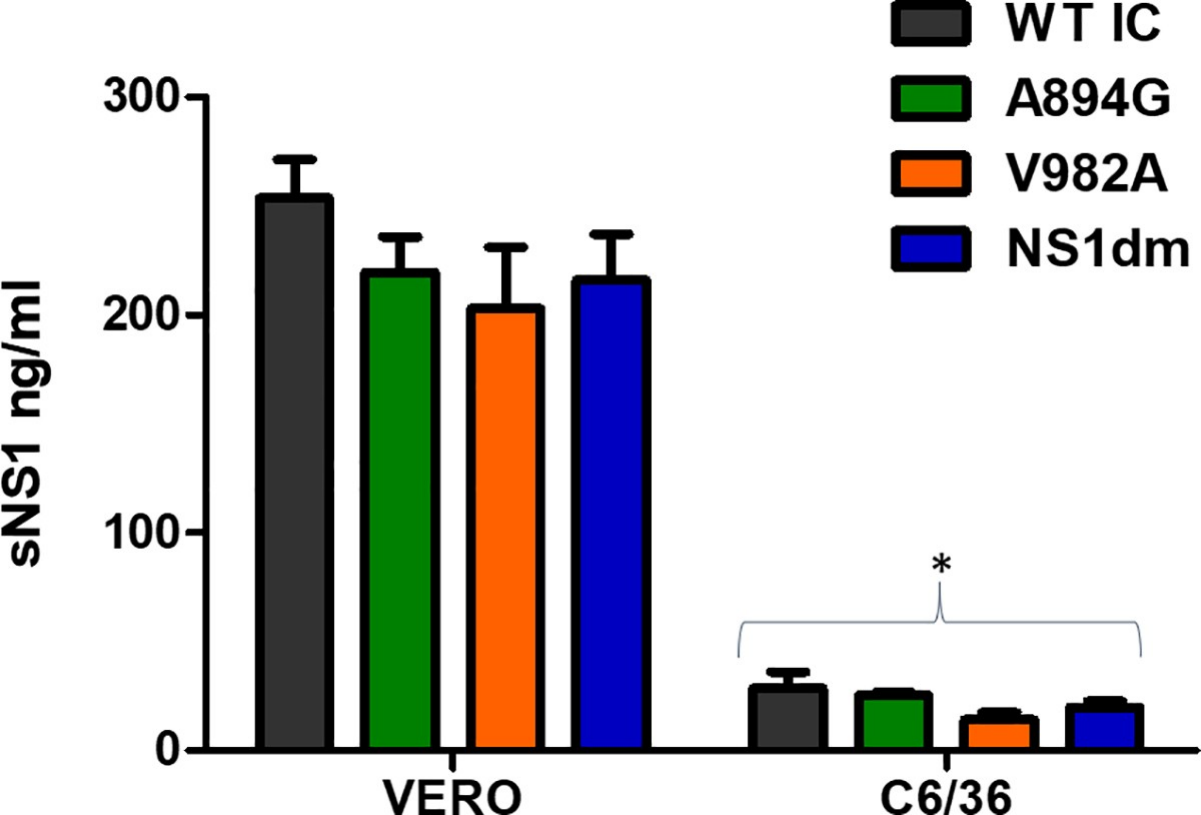
Levels of secreted Zika virus NS1 protein are similar among ZIKV strains. Wildtype and NS1 mutant strains of ZIKV were used to infect mammalian (Vero) and mosquito (C6/36) cells. Supernatants from the infected cell cultures were collected at peak titer in triplicate and sNS1 protein was quantified using ELISA. ZIKV sNS1 was identified in both cell lines, with significantly higher levels in mammalian cells (*two-way ANOVA, p<0.001). No statistically significant differences were identified among ZIKV strains or in interaction of strain and cell line (two-way ANOVA, p>0.05).

To test the hypothesis that NS1 mutations influence the levels of NS1 secretion, we measure the quantity of soluble NS1 protein (sNS1) in vertebrate and mosquito cells *in vitro*. sNS1 was quantified from Vero and C6/36 cell culture supernatants, respectively, using a ZIKV-specific ELISA. sNS1 was detected in supernatant of both vertebrate and invertebrate cells, although levels in vertebrate cells were significantly higher (Fig 6; two-way ANOVA, F (1,8) = 341.8, p<0.0001). No significant difference in sNS1 protein secretion in Vero or C6/36 cells were identified when comparing ZIKV-WTic to ZIKV- A894G, ZIKV-V982A or ZIKV-NS1dm in either cell line (Fig 6; two-way ANOVA, Sidak’s multiple comparisons-test, p>0.05). In addition, there was no interaction between cell line and strain (Fig 6; two-way ANOVA, p>0.05).

### *In vivo* infection kinetics

**Fig 7:**
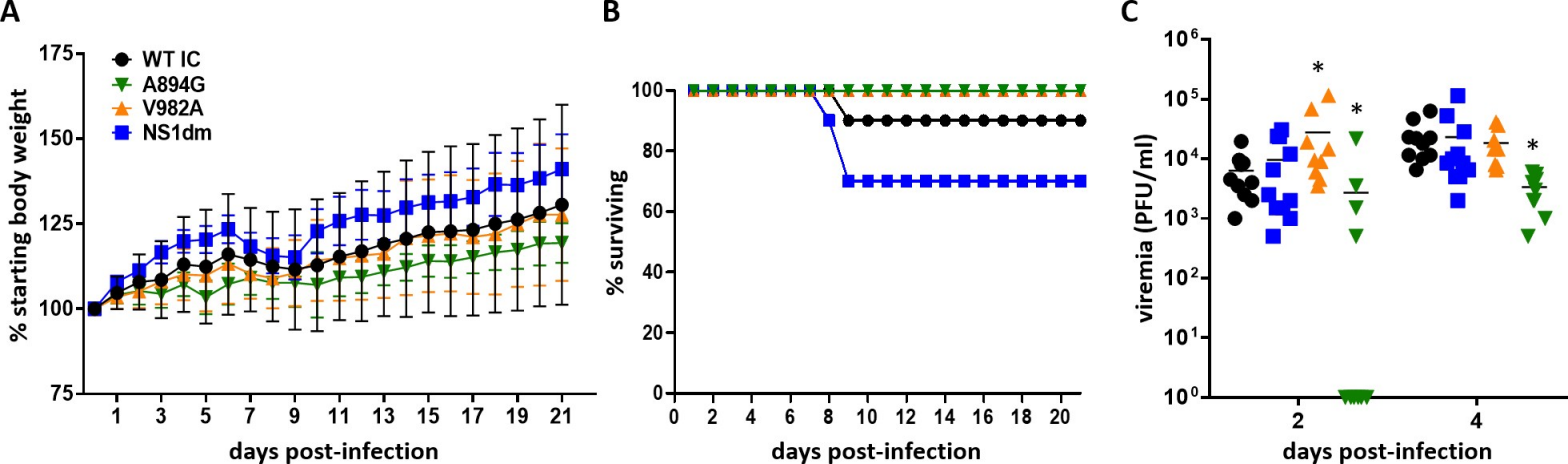
Characterization of wildtype Zika virus and NS1 mutants in *Ifnar1*^*-/-*^ mice. Groups of randomized, mixed sex three- to seven-week-old *Ifnar1*^*-/-*^ mice were subcutaneously inoculated with 10^3^ PFU of ZIKV-WTic and NS1 mutants (n = 8-11/strain). Mice were observed daily for 21 days and morbidity (A), mortality (B) and viremia (C) were measured. No significant differences in morbidity or mortality were identified. Strain had a significant impacted on viremia levels (Two-way ANOVA, p = 0.02). Viremia was significantly higher for ZIKV-V982A at 2 dpi compared to ZIKV-WTic, yet significantly lower for ZIKV-A894G at both 2 dpi and 4 dpi (*t-test, p<0.05). Viremia levels of ZIKV-NS1dm were equivalent to ZIKV-WTic at both 2 and 4 dpi (t-test, p>0.05).

To further analyze phenotypic differences between ZIKV-WTic and mutant strains of ZIKV *in vivo*, mouse infection experiments were conducted using mice with abrogated type I interferon signaling (*Ifnar1*^*-/-*^) that have been previously established for investigating flavivirus pathogenesis [32]. Groups of randomized, mixed sex three- to seven-week-old *Ifnar1*^*-/-*^ mice were subcutaneously inoculated in a hind footpad with 10^3^ PFU of ZIKV-WTic, ZIKV-NS1dm, ZIKV-V982A, or ZIKV-A894G. We collected serum samples at two and four days-post-inoculation (dpi) to confirm and compare viremia between groups. Mice were monitored daily for clinical signs for 21 days. Overt clinical signs were only evident in ZIKV-WTic- and ZIKV-NS1dm-inoculated animals and predominantly included hind limb paralysis. ZIKV-WTic, ZIKV-A894G, ZIV-V982A, and ZIKV-NS1dm survival curves did not differ significantly between groups (Fig 7B; Log-rank test, p = 0.12). Neither morbidity nor mortality was observed from the single mutant groups ZIKV-V982A or ZIKV–A894G. Strain had a significant impact on viremia levels (Fig 7C; Two-way ANOVA, F(3, 36) = 3.559, p = 0.02). Viremia was significantly higher for ZIKV-V982A at 2 dpi compared to ZIKV-WTic, yet significantly lower for ZIKV-A894G compared to ZIKV-WTic at both 2 dpi and 4 dpi (Fig 7C; t-test, p<0.05). Viremia levels of ZIKV-NS1dm were equivalent to ZIKV-WTic at both 2 and 4 dpi (t-test, p>0.05).

## Discussion

While our knowledge of ZIKV biology has vastly improved since its establishment in the Americas, the factors that facilitated its emergence and rapid dissemination, as well as the potential for expanded ZIKV transmission, are not fully understood. Specifically, the role of strain variation and/or adaptive evolution in ZIKV transmission remain unclear. In general, our results support the idea that U.S. *Ae*. *albopictus* are more competent for an ancestral Asian lineage strain and that this is largely a result of mutations in the NS1 gene. Contrasting these results with previous data [16] also suggests that epistatic interactions both within the NS1 and among NS1 and other genes could influence viral fitness in both vector and host.

The flavivirus NS1 is a multifunctional protein that exists in intracellular, membrane bound and secreted forms as a monomer, dimer and hexamer, respectively [33,34]. It has been shown to interact with each of the additional non-structural proteins as well as host proteins, and accordingly has been implicated in viral replication, secretion, and immune modulation [35,36]. NS1 secretion is a well-documented occurrence in vertebrate hosts contributing to a variety of functions including viral pathogenesis and virulence [36–39]. The fact that we identified free NS1 in C6/36 cell culture supports recent studies confirming that NS1 secretion can also readily occur in mosquito cells [34,40–46]. Youn et al. [47] observed that C6/36 cells infected with West Nile virus (WNV) did not secrete NS1 without two non-synonymous substitutions matching the DENV NS1, suggesting that species and strain-specific differences could facilitate the efficiency of NS1 secretion in mosquito cells. Further investigation is needed into the presence of sNS1 in mosquito hemolymph and its potential role in immunity and vector competence. This is particularly important given recent studies that clearly indicate a role for host-derived sNS1 in mosquito infectivity. Specifically, the presence of NS1 in blood meals has been shown to enhance infectiousness of DENV and Japanese encephalitis virus (JEV) in *Ae*. *aegypti* and *Cx*. *pipiens* by modifying mosquito immune responses [48]. Liu et al. demonstrated that A982V in the FSS13025 background [49] increased sNS1 levels and ZIKV competence of *Ae*. *aegypti* [16,50], which would predict that reversion to arginine at 982 would decrease competence of *Ae*. *aegypti*. The lack of strain-specific difference in *Ae*. *aegypti* infection measured in our study could be interpreted as a population-specific difference in susceptibility to sNS1, yet since we additionally did not demonstrate a significant difference in sNS1 levels in cell culture, our results further support a possible role for epistatic interactions with other mutations in the ancestral ZIKV background. Future studies evaluating the effect of NS1 mutations in multiple genetic backgrounds and diverse mosquito populations are needed in order to accurately assess both the role of epistasis and mosquito genetics in vector competence. In addition, although Liu et al demonstrated that relative differences in NS1 secretion in cell culture are reflective of differences *in vivo* [16], a comprehensive understanding of the effect of NS1 mutations on sNS1 levels will require thorough characterization of sera derived from hosts infected with diverse strains.

Given that A982V is the only shared amino acid mutation in the NS1 between pre- and post-epidemic Asian lineage strains, our results suggest that other genes or mechanisms may to some degree govern NS1 secretion and function. In addition to the two NS1 mutations investigated here, there are a total of 11 amino acid differences between FSS13025 and HND 2016, including three in the NS3 and two in the NS5. Future studies should evaluate how these and other residues in the nonstructural genes affect NS1 function and vector competence in general.

Given the lack of phenotypic effect in the primary vector measured here, results in mosquitoes would independently suggest that these NS1 mutations either have population-specific effects, were stochastically acquired in some genetic backgrounds, or are important in viral fitness in the host. Xia et al. indeed demonstrated that A982V enhances the capacity of ZIKV to evade the host interferon response by binding and reducing phosphorylation of TBK1 [50]. While this did not equate to differences in viremia in mice deficient in type-I and type-II interferon receptors (AG129), differences in ZIKV RNA levels in sera of immunocompetent C57/BL6J mice were measured. Importantly, this effect was reversed when reverting the 2015 Puerto Rican strain PRVABC-59 at this position. While we also utilized immunodeficient mice (type-I interferon deficient), it is notable that we measured the opposite effect, i.e. a modest increase in early viremia in ZIKV-V982A in the HND 2016 background. This result again points to the potential importance of host-specific differences and/or epistatic interactions in governing the relationship between NS1 mutations and viral fitness. This is perhaps less surprising in the host, as numerous genes including NS2, NS4 and NS5 have well documented roles in interferon modulation and host immunity in general [51–59]. The more phenotypically significant result in mice measured here was with ZIKV-A894G. Since viremia was significantly lower than ZIKV-WTic we speculate that the ancestral substitution of guanine to alanine at position 894 is likely an adaptive change which may have been acquired during circulation in the Americas to increase viral fitness in the host. Since the 894 substitution does not exist independent of the 982 substitution, it is plausible that this occurred to compensate for a fitness cost associated with A982V in this genetic background. This is further supported by the fact that ZIKV-NS1dm viremia was similar to ZIKV WTic.

Previous studies with CHIKV demonstrate that arbovirus adaptation to *Ae*. *albopictus* can occur without affecting competence in *Ae*. *aegypti* [60], indicating the potential for unique vector-virus interactions among these species. Our data support this and specifically suggest that ZIKV NS1 could differ in its interactions with *Ae*. *albopictus*. Since *Ae*. *albopictus* have not played a significant role in the global spread of ZIKV, it is perhaps not surprising that a transmission advantage in this species could be lost, yet these data demonstrate that strains with increased emergence potential in *Ae*. *albopictus* are likely currently in circulation and that adaptive evolution could further increase the vector range and transmissibility of ZIKV in the Americas.

## Supporting information

S1 Table Vector competence results from preliminary experiment comparing ZIKV WT-IC to ZIKV HND. (DOCX)

S2 Table Vector competence results from individual experiments comparing ZIKV WT-IC to NS1 mutants. (DOCX)
