# Supplementary material for: Reversion to ancestral Zika virus NS1 residues increases competence of Aedes albopictus
Source: PLoS Pathog. 2020 Oct 14;16(10):e1008951. doi: 10.1371/journal.ppat.1008951 (PMC7588074; doi:10.1371/journal.ppat.1008951)
Supplement: S1 Table — (DOCX) [file ppat.1008951.s001.docx]

**S1 Table. Vector competence results from preliminary experiment comparing ZIKV WT-IC to ZIKV HND.**

|  | | | **DAY 7** | | | | | | | **DAY 14** | | | | | | |
| --- | --- | --- | --- | --- | --- | --- | --- | --- | --- | --- | --- | --- | --- | --- | --- | --- |
| **species** | **virus** | **input^1^** | **exposed** | **infected** | **%** | **dissem^2^** | **%** | **trans^3^** | **%** | **exposed** | **infected** | **%** | **dissem^2^** | **%** | **trans^3^** | **%** |
| ***Ae. albopictus*** | **WT-IC** | **6.6** | **29** | **8** | **27.6** | **2** | **25.0** | **1** | **12.5** | **20** | **8** | **40.0** | **3** | **37.5** | **1** | **12.5** |
|  | **HND** | **6.8** | **24** | **10** | **41.2** | **3** | **30.0** | **0** | **0** | **24** | **11** | **45.8** | **4** | **36.4** | **1** | **9.1** |
| ***Ae. aegypti*** | **WT-IC** | **6.6** | **29** | **14** | **48.3** | **10** | **71.4** | **2** | **20.0** | **21** | **12** | **57.1** | **11** | **91.7** | **5** | **45.5** |
|  | **HND** | **6.8** | **30** | **15** | **50.0** | **12** | **80.0** | **4** | **25.0** | **29** | **16** | **55.2** | **13** | **81.3** | **5** | **38.5** |

^1^ log_10_ pfu ZIKV/ml blood

^2^ number of infected disseminated

^3^ number of infected transmitting
