# Supplementary material for: Reversion to ancestral Zika virus NS1 residues increases competence of Aedes albopictus
Source: PLoS Pathog. 2020 Oct 14;16(10):e1008951. doi: 10.1371/journal.ppat.1008951 (PMC7588074; doi:10.1371/journal.ppat.1008951)
Supplement: S2 Table — (DOCX) [file ppat.1008951.s002.docx]

**S2 Table.** **Vector competence results from individual experiments comparing ZIKV WT-IC to NS1 mutants.**

| **EXPERIMENT 1** | | | **DAY 7** | | | | | | | **DAY 14** | | | | | | |
| --- | --- | --- | --- | --- | --- | --- | --- | --- | --- | --- | --- | --- | --- | --- | --- | --- |
| **species** | **virus** | **input^1^** | **exposed** | **infected** | **%** | **dissem^2^** | **%** | **trans^3^** | **%** | **exposed** | **infected** | **%** | **dissem^2^** | **%** | **trans^3^** | **%** |
| ***Ae. albopictus*** | **WT-IC** | **6.8** | **29** | **10** | **34.5** | **3** | **30.0** | **1** | **12.5** | **20** | **10** | **50.0** | **7** | **70.0** | **1** | **10.0** |
|  | **NS1DM** | **6.6** | **30** | **19** | **63.3** | **9** | **47.4** | **1** | **5.3** | **20** | **18** | **90.0** | **8** | **44.4** | **1** | **5.6** |
| ***Ae. aegypti*** | **WT-IC** | **6.8** | **21** | **12** | **57.1** | **9** | **75.0** | **1** | **8.3** | **21** | **14** | **66.6** | **14** | **100.0** | **7** | **50.0** |
|  | **NS1DM** | **6.6** | **31** | **19** | **61.2** | **11** | **57.8** | **1** | **5.3** | **27** | **15** | **55.5** | **12** | **80.0** | **4** | **26.6** |
| **EXPERIMENT 2** | | |  |  |  |  |  |  |  |  |  |  |  |  |  |  |
| ***Ae. aegypti*** | **NS1DM** | **6.6** | **NC** | **NC** | **NC** | **NC** | **NC** | **NC** | **NC** | **30** | **17** | **56.6** | **15** | **88.2** | **6** | **35.2** |
|  | **V982A** | **6.9** | **NC** | **NC** | **NC** | **NC** | **NC** | **NC** | **NC** | **30** | **18** | **60.0** | **14** | **77.8** | **6** | **33.3** |
| **EXPERIMENT 3** | | |  |  |  |  |  |  |  |  |  |  |  |  |  |  |
| ***Ae. albopictus*** | **WT-IC** | **7.2** | **30** | **13** | **43.3** | **6** | **46.1** | **0** | **0.0** | **30** | **20** | **66.6** | **17** | **85.0** | **3** | **15.0** |
|  | **NS1DM** | **6.7** | **30** | **24** | **80.0** | **8** | **33.3** | **0** | **0.0** | **30** | **24** | **80.0** | **18** | **75.0** | **3** | **12.5** |
|  | **A894G** | **7.2** | **26** | **20** | **76.9** | **12** | **60.0** | **4** | **20.0** | **24** | **19** | **79.2** | **17** | **89.5** | **11** | **57.9** |
|  | **V982A** | **7.0** | **30** | **23** | **76.7** | **12** | **52.2** | **0** | **0.0** | **30** | **26** | **86.7** | **26** | **100.0** | **7** | **26.9** |
| ***Ae. aegypti*** | **WT-IC** | **7.2** | **30** | **22** | **73.3** | **20** | **90.9** | **2** | **9.1** | **30** | **23** | **76.6** | **23** | **100.0** | **16** | **66.7** |
|  | **NS1DM** | **6.7** | **30** | **22** | **73.3** | **20** | **90.1** | **1** | **4.5** | **30** | **24** | **80.0** | **23** | **95.8** | **9** | **39.1** |
|  | **A894G** | **7.2** | **30** | **24** | **80.0** | **19** | **80.0** | **6** | **30.0** | **28** | **23** | **80.0** | **23** | **100.0** | **14** | **60.0** |
|  | **V982A** | **7.0** | **30** | **27** | **90.0** | **27** | **100.0** | **0** | **0.0** | **28** | **24** | **85.7** | **23** | **95.8** | **12** | **50.0** |

NC = not completed

^1^ log_10_ pfu ZIKV/ml blood

^2^ number of infected disseminated

^3^ number of infected transmitting
